# Factors associated with caries: a survey of students from southern Brazil

**DOI:** 10.1016/j.rppede.2016.02.013

**Published:** 2016

**Authors:** Tássia Silvana Borges, Natalí Lippert Schwanke, Cézane Priscila Reuter, Léo Kraether Neto, Miria Suzana Burgos

**Affiliations:** Universidade de Santa Cruz do Sul (UNISC), Santa Cruz do Sul, RS, Brazil

**Keywords:** Dental caries, Epidemiology, Students

## Abstract

**Objective:**

To describe the factors associated with dental caries among students from Santa Cruz do Sul, Rio Grande do Sul, Brazil.

**Methods:**

A cross-sectional study was conducted in a random sample of 623 students of both genders, aged 10-17 years old. Tooth decay was performed using the index of the World Health Organization (1997), DMFT (permanent dentition) that expresses the sum of decayed, missing and filled teeth per person. The maternal educational level was rated using criteria of the Brazilian Association of Market Research Companies. The remaining variables were obtained by a structured questionnaire. Poisson regression analysis was used to test the association between variables using robust models and a subsequently adjusted model. Data were expressed as prevalence ratio (PR).

**Results:**

Multivariate analysis identified the following factors related to the experience of dental caries: residence in rural municipalities (PR: 1.15; 95%CI: 1.0-1.3), attending a city school (PR: 3.30; 95%CI: 1.1-9.4) or a state school (PR: 3.40; 95%CI: 1.1-9.6); and having an illiterate mother or a mother that only attended up to the 4th year of school (PR: 1.67; 95%CI: 1.1-2.4) or high school (PR: 1.54; 95%CI: 1.1-2.2).

**Conclusions:**

The presence of caries in students in southern Brazil was associated with residence in rural areas, mother with little education and attendance to a public school.

## Resumo

**Objetivo:**

Descrever os fatores associados à cárie dentária em escolares de Santa Cruz do Sul, Rio Grande do Sul, Brasil.

**Métodos:**

Um estudo transversal foi feito em uma amostra aleatória de 623 estudantes de ambos os sexos, entre 10 e 17 anos. A cárie dentária foi avaliada com o índice da Organização Mundial de Saúde (1997) CPO-D (dentição permanente), que constitui a soma de dentes cariados, perdidos e obturados por pessoa. A escolaridade materna foi avaliada com os critérios da Associação Brasileira de Empresas de Pesquisa. As demais variáveis foram obtidas com um questionário estruturado. A análise de regressão de Poisson foi usada para testar a associação entre as variáveis, com modelos consistentes e um modelo ajustado posteriormente. Os dados foram expressos como razão de prevalência (RP).

**Resultados:**

A análise multivariada identificou os seguintes fatores relacionados à experiência de cárie dentária: residência em municípios rurais (RP: 1,15; IC95%: 1,0-1,3), frequentar uma escola municipal (RP: 3,30; IC95%: 1,1-9,4) ou estadual (RP: 3,40; IC95%: 1,1-9,6); e ter uma mãe analfabeta ou uma mãe que só estudou até a 4ª série do Ensino Fundamental (RP: 1,67; IC95%: 1,1-2,4) ou o ensino médio (RP: 1,54; IC95%: 1,1-2,2).

**Conclusões:**

A presença de cárie em alunos no sul do Brasil foi associada com residência em áreas rurais, mãe com baixo nível educacional e frequentar escola pública.

## Introduction

According to the World Health Organization (WHO), the prevalence of dental caries among schoolchildren is 90% in some countries.^1^ In Brazil, the prevalence in children is 53.4% and in adolescents, 56.5%.^2^ Dental caries is one of the most researched oral diseases^3^^,^ 4 and results from a chronic process that occurs after a certain amount of exposure to a cariogenic diet and to tooth-susceptible microorganisms. Caries is considered a multifactorial condition that is transmitted locally and is one of the most prevalent pathologies in childhood.^5^^,^ 6

Risk factors for dental caries include salivary flow and composition, cariogenic bacteria present, inadequate fluoride exposure, immune components and genetic factors.^7^^-^ 9 However, other factors, such as lifestyle, behavior, hygiene, eating habits, social status and sociodemographic factors, also contribute to the evolution of caries.^6^^,^ 10 ^-^ 12 Oral diseases affect daily activities; among these activities, increased absenteeism^13^ and decreased performance at school and work have widespread economic and psychological impacts and can lead to significant reductions in individual quality of life.^14^^,^ 15 In this context, this study aimed to describe factors associated with caries among students in Santa Cruz do Sul, Rio Grande do Sul, Brazil.

## Method

A cross-sectional study of 623 students of both sexes, aged 10-17 years old, was conducted from April to December 2012. The sample consisted of students from public and private schools in the city of Santa Cruz do Sul, Rio Grande do Sul, Brazil.

Santa Cruz do Sul has a population of 118,374 inhabitants, according to the census of the Brazilian Institute of Geography and Statistics (IBGE).^16^ In the urban area of the municipality there are 105,190 inhabitants, and in the rural area, 13,184 inhabitants. The Human Development Index (HDI) is 0.773. The city currently has 99% of households served by drinking water with adequate fluoridation. The municipality has 64 establishments of the Unified Health System (SUS), 12 Basic Health Units (UBS) (4 in the rural area and 8 in the urban area) and, among them, six have Surgeon Dentists. Ten teams are dedicated to the Family Health Strategy (1 in the rural area and 9 in the urban area) and four of them have oral health team. In 2012, 14,899 registrations were made in primary education (1751 in private schools, in 6958 city public schools, and 6190 in state public schools). In high school, there were 4010 registrations (765 in private schools and 3245 in public schools).^17^

The reference population for the study consisted of approximately 20,540 students in primary and high schools in the public and private school network of the city of Santa Cruz do Sul, stratified by rural vs. urban areas and, further, by center and periphery: north, south, east or west. The city has a total of 69 schools: eight are private schools, all of them located in the urban area, 30 are city schools ans 31 municipal schools. In the rural area, there are six state schools and 16 city schools.^17^ In order to calculate the sample size, Epi-Info program (Centers for Disease Control and Prevention, Atlanta, GA, USA, version 7) was used. The prevalence of tooth decay used for the calculation was 56%,^2^ with 80% power and a standard error of 5%. Based on these parameters, the minimum required sample size was 362 students. When the confounding variables were added to control the effect by 20% and 20% for losses and refusals, the required sample was 522 students.

The inclusion criteria were: informed consent form signed by a parent/guardian; the adolescent was willing and able to conduct an examination of the oral cavity; the adolescent was properly enrolled in the school, and the adolescent was within the age range of 10-17 years. Both genders were included in the study. The adolescents were excluded from the study if they were absent from school at the time of the oral health evaluation or if they did not permit the evaluation or if they did not complete the questionnaires properly. Two attempts of assessment of oral health per student were carried out.

To evaluate dental caries, an examiner conducted the calibration initially, and the kappa test revealed a correlation of *K*=0.90. Calibration was performed between the examiner of the study and a researcher with extensive prior experience. The assessment of oral health was performed at the university's research laboratory by a previously trained researcher. The assessment followed the protocol of the SB Brazil Project.^2^ During the oral examination, the student and the researcher remained seated in chairs in front of a window in order to obtain the maximum natural lighting. The mean duration of the dental examination was 10min. The test was conducted with a WHO *ballpoint* probe and a number 5 dental mirror, all packed in surgical and autoclaved paper. No radiography was performed, emphasizing that no pre-drying and brushing or prophylaxis of the teeth were performed before the examination. All the codes and criteria were recorded on individual records for each student. The index used was the DMFT (decayed, missing and filled/restored teeth) index. The DMFT index measures, in a specified population, the average number per person of: decayed permanent teeth in need of a filling or extraction (D), missing permanent teeth that have been removed as a result of caries (M), and filled permanent teeth (F). Sometimes, the separate components of the DMFT index are used as a measure of service utilization (e.g., the F component is an indication of dental treatment of decayed teeth), and the M component may suggest what type of dental care has or has not been received (i.e., a high M component suggests that teeth were extracted as a result of untreated decay).

The maternal education level was assessed using a questionnaire adapted with the criteria of the Brazilian Association of Market Research Companies (Associação Brasileira de Empresas de Pesquisa - ABEP).^18^ Three categories were used: illiterate or up to the 4th year of school, high school education, and higher education. Age of the students was defined by the WHO^19^ criteria as follows: early adolescence (10-13 years), middle adolescence (14-16 years) and late adolescence (17-19 years).

Oral hygiene was assessed with a questionnaire adapted from Barros and Nahas,^20^ with questions related to the frequency of flossing and tooth brushing. We also used a self-administered questionnaire on dietary habits proposed by Barros and Nahas,^20^ with questions related to the frequency of consumption of sweet foods, snacks and soft drinks.

Data analysis was performed using Statistical Package for the Social Sciences (SPSS) software version 20.0 for Windows (IBM Corp. Released 2011, Armonk, NY: IBM Corp, USA). The significance level used was 5%. Data were described by absolute and relative percentages. Chi-square tested the association of dental caries with other variables. Poisson regression analysis was applied to verify the association between the dependent (DMFT≥1) and independent variables using robust models; for these models, significance levels of *p*<0.05 or *p*<0.20 were adopted. The adjusted model included the variables that were considered significant (*p*<0.05).

The research project was submitted and approved by the Ethics Committee in Research of the University of Santa Cruz do Sul, under the protocol number 3044/11, in accordance with the Declaration of Helsinki. All students who participated in the study had informed consent forms on record signed by their parents or guardians.

## Results

In total, 623 students with a mean age of 13.5 years were evaluated; among these students, 57.9% were female, 50.1% lived in the urban area, 65.3% studied in state schools, and 59.2% had mothers who were illiterate or who had four or less years of schooling. The students reported consuming sweets and soft drinks 2-6 times per week, and they most frequently consumed chips once per week. Regarding students' oral hygiene habits, 83.1% brushed their teeth 1-3 times per day, and flossing frequency was reported as “sometimes” by 52.0% of the students. Dental caries showed positive associations with area of residence, type of school, maternal level of education and frequency of flossing. The mean DMFT index was 2.5 (Table 1), which means that each child had an average of 2.5 decayed or lost or restored teeth in the mouth at the time of evaluation.

**Table 1:** Characterization of students (*n*=623).

	Total *n* (%)	DMFT≥1 *n* (%)	DMFT=0 *n* (%)	*p*-value^a^
*Sex*				0.204
Female	361 (57.9)	237 (65.7)	124 (34.3)	
Male	262 (42.1)	159 (60.7)	103 (39.3)	

*Housing area*				<0.001
Urban	312 (50.1)	176 (56.4)	136 (43.6)	
Rural	311 (49.9)	220 (70.7)	91 (29.3)	

*School type*				<0.001
Particular	22 (3.5)	03 (13.6)	19 (86.4)	
State	407 (65.3)	259 (63.6)	148 (36.4)	
Municipal	194 (31.1)	134 (69.1)	60 (30.9)	

*Mother's educational level*				<0.001
Completed college	57 (9.1)	19 (33.3)	38 (66.7)	
High school	197 (31.6)	119 (60.4)	78 (39.6)	
Illiterate or up to the 4th grade	369 (59.2)	258 (69.9)	111 (30.1)	

*Consumption of sweets*				0.678
1-3 times per day	141 (22.6)	95 (67.4)	46 (32.6)	
2-6 times per week	237 (38.0)	151 (63.7)	86 (36.3)	
1 time per week	213 (34.2)	131 (61.5)	82 (38.5)	
Never	32 (5.1)	19 (59.4)	13 (40.6)	

*Snack consumption*				0.092
1-3 times per day	88 (14.1)	59 (67.0)	29 (33.0)	
2-3 times per day	210 (33.7)	144 (68.6)	66 (31.4)	
1 time per week	270 (43.3)	164 (60.7)	106 (39.3)	
Never	55 (8.8)	29 (52.7)	26 (47.3)	

*Soft drink consumption*				0.375
1-3 times per day	142 (22.8)	89 (62.7)	53 (37.3)	
2-6 times per week	248 (39.8)	167 (67.3)	81 (32.7)	
1 time per week	211 (33.9)	128 (60.7)	83 (39.3)	
Never	22 (3.5)	12 (54.5)	10 (45.5)	

*Frequency of brushing*				0.524
1-3 times per day	518 (83.1)	325 (62.7)	193 (37.3)	
4-5 times a day	87 (14.0)	60 (69.0)	27 (31.0)	
1-3 times per week/no brushing	18 (2.9)	11 (61.1)	07 (38.9)	

*Flossing*				0.002
Yes/daily	154 (24.7)	109 (70.8)	45 (29.2)	
Sometimes	324 (52.0)	185 (57.1)	139 (42.9)	
Never	145 (23.3)	102 (70.3)	43 (29.7)	

DMFT≥1, decayed, missing and filled/restored teeth; DMFT=0: school caries free.

a *p-*value, Chi-square test.

As shown in Table 2, students living in the rural area of the municipality were 25% more likely to develop dental caries (DMFT≥1) compared with those living in the urban area. Students attending municipal or State schools had a five-time greater likelihood of developing dental caries compared with those from private schools. Students with mothers who completed no more than the 4th year of schooling were two times more likely to develop dental caries compared with children of mothers who had more education. Nevertheless, students with mothers who had completed high school had an approximately 81% more chance of developing tooth decay compared with students whose mothers had completed higher education. Regarding oral hygiene habits, flossing “sometimes” had a 20% protective effect compared on the likelihood of caries, compared to not flossing at all.

**Table 2:** Associations between the DMFT≥1 index and the following specific variables: housing area, school type, mother's educational level, dietary habits and oral hygiene habits (brushing and flossing frequencies).

Variable	Crude PR (95%CI)	*p*-value^a^
*Housing area*		
Urban	1	
Rural	1.25 (1.1-1.4)	<0.001

*School type*		
Municipal	5.06 (1.7-14.5)	0.003
State	4.66 (1.6-13.3)	0.004
Particular	1	

*School education of mother*		
Illiterate or up to the 4th grade	2.09 (1.4-3.0)	<0.001
High school	1.81 (1.2-2.6)	0.002
Completed college	1	

*Frequency of brushing*		
1-3 times per week/no brushing	0.97 (0.6-1.4)	0.9
4-5 times per day	1.09 (0.9-1.2)	0.2
1-3 times per day	1	

*Flossing*		
Yes/daily	1.00 (0.8-1.1)	0.9
Sometimes	0.81 (0.7-9.3)	0.004
Never	1	

*Consumption of sweets*		
1-3 times per day	1.13 (0.8-1.5)	0.5
2-6 times per week	1.03 (0.7-1.4)	0.8
1 time per week	1.07 (0.7-1.4)	0.6
Never	1	

*Snack consumption*		
1-3 times per day	1.27 (0.9-1.6)	0.1
2-6 times per week	1.15 (0.8-1.5)	0.4
1 time per week	1.30 (0.9-1.6)	0.5
Never	1	

*Soft drink consumption*		
1-3 times per day	1.14 (0.7-1.7)	0.5
2-6 times per week	1.11 (0.7-1.6)	0.6
1 time per week	1.23 (0.8-1.8)	0.3
Never	1	

PR, prevalence ratio; CI, confidence interval.

a Poisson regression with robust (crude) models.

Table 3 presents the analysis adjusted for confounding factors. Living in a rural area, having a mother with a lower education level and attending a municipal or state public school were independently associated with the presence of dental caries.

**Table 3:** Associations between DMFT≥1 and other variables: area of residence, type of school, mother's educational level and oral hygiene habits, adjusted for sex and age.

Variable	Adjusted PR (95%CI)	*p*-value^a^
*Housing area*		
Urban	1	
Rural	1.15 (1.0-1.3)	0.030

*School type*		
Municipal	3.30 (1.1-9.4)	0.026
State	3.40 (1.1-9.6)	0.022
Particular	1	

*School education of mother*		
Illiterate or up to the 4th grade	1.67 (1.1-2.4)	0.005
High school	1.54 (1.0-2.2)	0.023
Completed college	1	

*Flossing*		
Yes/daily	0.97 (0.8-1.1)	0.703
Sometimes	0.80 (0.7-0.9)	0.003
Never	1	

PR, prevalence ratio; CI, confidence interval.

a Poisson regression with robust adjusted models.

## Discussion

This study demonstrated independent associations between students' DMFT ≥indices and sociodemographic variables such as: living in rural areas, attending municipal or state public schools and being the children of mothers with less schooling.

Descriptive analyses of the mean DMFT (2.5) index indicated good oral health among this population when compared to the Brazilian context. Data from the national oral health survey conducted in 2010 showed that the DMFT index at 12 years old was 2.06; at 15-19 years old, 4.01.^2^ For World Health Organization, this result is considered of low severity, being within the range of 1.2-2.6,^21^ which means that on average, each child has 1.2-2.6 teeth decayed, missing or extracted in mouth.

Students living in rural areas had a greater likelihood of developing dental caries compared with students of urban areas. Studies conducted in Greece,^22^^,^ 23 Australia,^24^ Pakistan^25^ and Brazil^26^^,^ 27 showed a higher prevalence of dental caries and treatment needs in schoolchildren from rural areas. This result could be strongly associated with the poor health status and the limited health services access of the inland population, which emphasizes the urgency of focusing attention on these areas to supply their residents with health information and with teams of professionals who are trained to meet their needs.^26^

In terms of the type of school, public school students were approximately five times more likely to develop dental caries compared with students from private schools. Similarly, a study conducted in Porto Alegre, Brazil,^28^ showed that students from the public school system came from families with lower education levels and that students from the private school network came from families with higher incomes. In our study, approximately 59.2% of the students' mothers were illiterate or had at most completed only the 4th year of school, in contrast with 9.1% of students whose mothers had completed higher education.

Several studies in the literature have addressed the relationship between the state of oral health with lower income and, consequently, with less educated parents. In most cases, limited access to information and health-related negative behaviors are associated with these findings and special attention should be given to this group.^12^^,^ 29 ^-^ 32 In our study, regarding oral hygiene, most of the schoolchildren performed daily tooth brushing (1-3 times), but 2.9% reported brushing their teeth 1-3 times per week or not brushing their teeth at all. As for flossing, most of the schoolchildren reported using dental floss only sometimes. Poor oral hygiene practices are directly related to oral health conditions, particularly to the development of dental caries.^33^

A study conducted in Thailand with 1156 students with 6 years old and 1116 students with 12 years old demonstrated a positive association between tooth decay development and poor oral hygiene practices^34^ Research conducted in Clementina and Gabriel Monteiro, in Brazil, showed that the sooner children began good oral hygiene practices, the lower the prevalence of early dental caries were.^35^

The limitations of this study are inherent to cross-sectional studies, which do not allow establishing a relationship of cause and effect. However, it must be noted that that the sample was representative of the studied municipality, with random selection of all students from public and private schools, urban areas and rural areas. The results of this study can only be generalized to populations with characteristics similar to the studied adolescents.

In summary, this study demonstrated that students residing in rural areas, students of state and city schools, and students of mothers with less schooling experienced a DMFT≥1. Moreover, direct action aimed at implementing educational and preventive programs on the topics of hygiene and self-care is necessary, particularly for parents and school communities in rural areas.
